# Inventory of landslides triggered by an extreme rainfall event in Marche-Umbria, Italy, on 15 September 2022

**DOI:** 10.1038/s41597-023-02336-3

**Published:** 2023-07-03

**Authors:** M. Santangelo, O. Althuwaynee, M. Alvioli, F. Ardizzone, C. Bianchi, T. Bornaetxea, M. T. Brunetti, F. Bucci, M. Cardinali, M. Donnini, G. Esposito, S. L. Gariano, S. Grita, I. Marchesini, M. Melillo, S. Peruccacci, P. Salvati, M. Yazdani, F. Fiorucci

**Affiliations:** 1 Italian National Research Council - Research Institute for Geo-Hydrological Protection (CNR-IRPI), Perugia, Italy; 2 Universidad del País Vasco/Euskal Herriko Unibertsitatea (UPV/EHU) (visiting at CNR-IRPI), Leioa, Spain; 3 Università degli Studi di Roma “La Sapienza”, Rome, Italy

**Keywords:** Natural hazards, Environmental impact

## Abstract

Systematic and timely documentation of triggered (i.e. event) landslides is fundamental to build extensive datasets worldwide that may help define and/or validate trends in response to climate change. More in general, preparation of landslide inventories is a crucial activity since it provides the basic data for any subsequent analysis. In this work we present an event landslide inventory map (E-LIM) that was prepared through a systematic reconnaissance field survey in about 1 month after an extreme rainfall event hit an area of about 5000 km^2^ in the Marche-Umbria regions (central Italy). The inventory reports evidence of 1687 triggered landslides in an area of ~550 km^2^. All slope failures were classified according to type of movement and involved material, and documented with field pictures, wherever possible. The database of the inventory described in this paper as well as the collection of selected field pictures associated with each feature is publicly available at figshare.

## Background & Summary

Landslides are widespread natural phenomena that can cause severe damage to structures, activities, and human life^1–4^. Landslides caused by triggering events are referred to as event landslides^5,6^. A triggering event can generate single landslides or tens of thousands of landslides^7–9^ and as such the affected areas span from single slopes to entire regions^5,10,11^. The most common landslide triggering events are meteo-climatic^1,11–15^ or seismic^10,16–19^, but landslides can be also induced by volcanic eruptions or human activity^2^.

Landslide inventory maps (LIMs), the simplest tool to represent landslide spatial distribution, register the location and type of landslides in an area^7^. If available, information on age, material involved, damage, and remediation works^20^ may be collected depending on the purpose of the survey. Usually, landslides are reported as area features (polygon shapes)^20–22^, but, depending on the scale of the map, lines or points features may be used^20^. A LIM that reports the landslides triggered by a specific triggering event is referred to as event landslide inventory map (E-LIM^7,8,23,24^,). E-LIMs are a valuable source of information as they report and depict the ground effects (in terms of landslides) of a triggering event. In a changing climate, systematic collection of such data is crucial^25^. E-LIMs also provide key information for the validation of landslide susceptibility models, and for hazard and risk analyses^7,26^.

Several methods can be used to map event landslides, including reconnaissance field survey^27^ expert interpretation of optical^5,21^ or SAR images^6,15^, automatic^28,29^ and semi-automatic classification algorithms^30,31^. However, data extraction from optical images strongly depends on cloud cover, while the capability of SAR images for event landslide inventory making is still underexplored^6^. Thus, especially for meteo-climatic events, reconnaissance field survey remains a very useful approach, due to its independence on favourable weather conditions. Also, field-based E-LIMs can provide very detailed information on even very small landslides under the trees canopy, which would likely remain unnoticed if aerial images alone are used. On the other hand, E-LIMs prepared by field activities often suffer from (i) incompleteness and (ii) a larger degree of inaccuracy compared to both expert and (semi-)automatic techniques. The first (i.e., incompleteness) is primarily due to the limitations of the visibility in the field^32^, accessibility of private locations, and road blockages which reduces the amount of territory that can be effectively observed. Furthermore, event inventory maps can be all the more complete the closer data is collected to the date of the event, regardless of the method used. Vegetation regrowth, erosion, and damage restoration activities may partially or totally cancel the evidence of (small to medium size) event landslides, even a few days after the triggering event. The second (i.e., inaccuracy) is due to the manual drawing of the landslides observed in the field on a geographic map (^24,27^). Besides, as for any measure requiring human operations, landslide inventories can be affected by systematic and accidental errors, and their overall quality also depends on the experience and skills of the geomorphologist(s). It has also been shown that field-based E-LIMs are affected by a larger error (especially in terms of geographic accuracy and completeness) than those produced by remote sensing techniques, which are considered as benchmarks in comparative studies^24,27^.

**Fig. 1:**
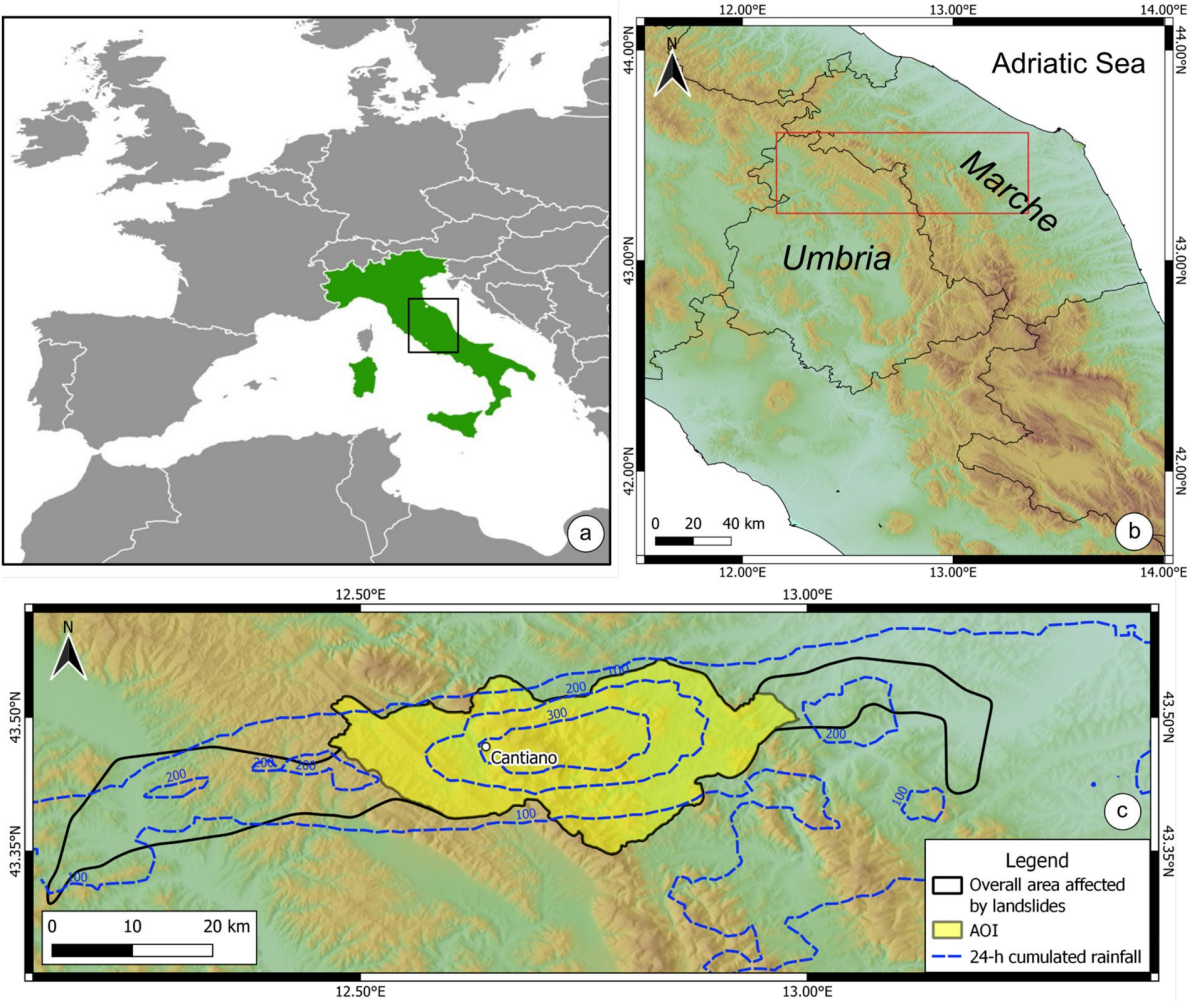
Location map. (**a,b**), geographic location of the area of interest. (**c**) Spatial arrangement of (i) the 24-h cumulated rainfall (i.e., isohyets, dashed blue lines), (ii) the overall area affected by landslides (black outlined polygon), (iii) the area where the event landslide inventory map was prepared (Area of Interest, AOI, yellow polygon). Location of the Cantiano rain gauge is provided.

This paper describes the E-LIM produced to record the landslides triggered by the extreme rainfall event that hit Marche and Umbria regions, in central Italy, on 15th September 2022. The rainfall event hit an area of ~5,000 km^2^ (Fig. 1), with peak rainfall intensity of 419 mm in 9 hours (Cantiano rain gauge, Fig. 1b), an exceptionally intense rainfall for this area, where the maximum recorded rainfall intensity was 120 mm in 9 hours or 173 mm in 12 hours. The rainfall record shows no significant rainfall in the 30-day period preceding the event: around 100 mm maximum were measured between August 15 and in September 1, and only a few mm in the first 15 days of September. This suggests dry soil conditions in the 15-day period preceding the event, also given the summer season. The event generated widespread floods and landslides. As a direct consequence of floods and landslides, many roads were interrupted, extensive damage was recorded to structures and infrastructures and to human life (11 deaths and 1 missing person). The E-LIM presented is the result of an extensive reconnaissance field survey covering a large neighbourhood of the area affected by the highest rainfall intensity^33^ (yellow polygon in Fig. 1c).

## Methods

**Fig. 2:**
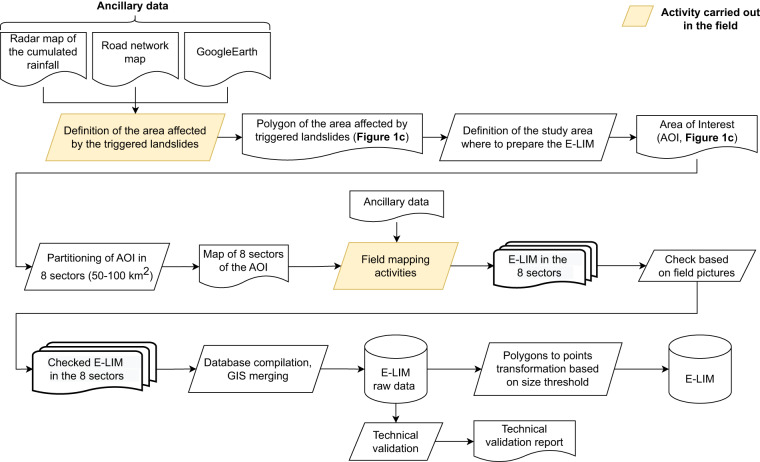
Flow chart of the procedure to prepare the E-LIM presented in this paper.

In this section we illustrate in detail the methods adopted to prepare the field-based E-LIM of the Umbria-Marche landslide event (available for download at: 10.6084/m9.figshare.21981842^34^). Figure 2 provides an overview of the activities carried out to prepare the E-LIM.

To carry out the field activities we used the following data as ancillary materials:

- Radar rainfall estimates provided by the National Department of Civil Protection (https://radar.protezionecivile.it/);
- Road network by OpenStreetMap^35^;
- Google Earth base map imagery.

Furthermore, the following tools were used^36^:

- Equipments:
  - Binoculars;
  - GPS receivers;
  - Laptops;
  - Off-road vehicles;
  - Inverter;
  - Smartphones;
  - Cameras with high optical zoom capability (42×) and geotagging.
- Software:
  - Google Earth (mapping in the field);
  - QGIS (for post-processing, QGIS Development Team, 2009);
  - ArcGIS 10^37^ (for topological check in the technical validation, Redlands, 2011);
  - GRASS GIS^38^

The first preparatory activity consisted in the definition of the area affected by the triggered landslides. A rapid extensive reconnaissance field survey was carried out driving and stopping at key scenery points within the broad area hit by the rainfall event (Fig. 2). During the survey, landslides were quickly reported by placemarks in a map wherever landslides could be recognised. According to this activity, the overall area affected by the landslide event is ~970 km^2^ (black outlined polygon in Fig. 1c). In addition, the preliminary field observations allowed us also to collect information about the main landslide types that were triggered. Following the classification by Hungr *et al*., (2014), a legend was defined according to such observations (Table 1) to ease, guide and homogenise the classification of landslides reported in the inventory.

**Table 1:** Description of the landslide types included in the E-LIM.

Type	Description
Debris/Earth/Rock Slide	Slide type landslides present a well-defined scarp and are commonly translational movements. The slide deposit is convex and the material is characterised by low mobility. This type of landslide involves mainly earth and debris and occasionally rock material.
Debris/Earth Slide-flow	Slide-flow initiates as a slide, then evolves into flow. Therefore, they show the characteristics of slides (most commonly translational) in the escarpment area but the deposit is elongated and sometimes branched due to the high water content. Material involved in this type of movement is debris and earth
Debris/Earth Flow	Flow landslides involve the movement of material down a slope in the form of a fluid. Flows often leave behind a distinctive, upside-down funnel shaped deposit where the landslide material has stopped moving. There are different types of flows:earth and debris.
Rock Fall	Falls are landslides that involve the collapse of material from a steep slope or a cliff. A fall-type landslide results in the collection of rock or debris near the base of a slope.
Widespread earth slide/debris slide/ debris flow	Widespread landslides indicate areas affected by clustered (often coalescent) slope failures so that they cannot be singled out.

We then selected a smaller area - defined with morphological criteria (following ridges and valleys) - within the overall area affected by the landslide event where to prepare the E-LIM (area of interest, AOI, yellow polygon in Fig. 1c). The perimeter of the AOI has been defined so as to encompass contiguous areas affected by landslides widespread in the landscape, including road slopes (cut and fill), cultivated areas, and natural slopes. This condition does not exist outside the AOI, where landslides occurrence was limited to road cut and artificial slope-break in farmlands, and only occasionally in the natural landscape. Visual inspection of Fig. 1c reveals that the AOI defined as said before, is roughly centred around the recorded rainfall peak, where the event was more intense and landslides impact severe. The AOI extends for 550 km2, i.e. 56.7% of the overall area affected by landslides. Such definition ensured that the inventory is representative of landslides types and sizes triggered by the event, as well as of the type of slopes affected by landslides (i.e. natural or artificial). Furthermore, in the AOI the distribution of land use and lithology is comparable to the distribution in the broader area affected by landslides. The AOI was then subdivided into 8 sectors of ~50–100 km^2^ each, to optimise the management of field activities (Fig. 2). Each sector was assigned to a mapping team to avoid duplications and improve logistics.

The reconnaissance field survey was carried out by five teams, each led by a geomorphologist expert in landslide mapping. Each team drove and walked along main and secondary roads taking note of location, area and classification of all detected landslides reported (on site) on Google Earth. Whenever possible, pictures of landslides were taken from any available point of view. Overall, the activities for the preparation of the E-LIM were carried out in a time interval of 33 days (from 22/09/2022 to 24/10/20223) and required a total of 12 days of field activities.

**Fig. 3:**
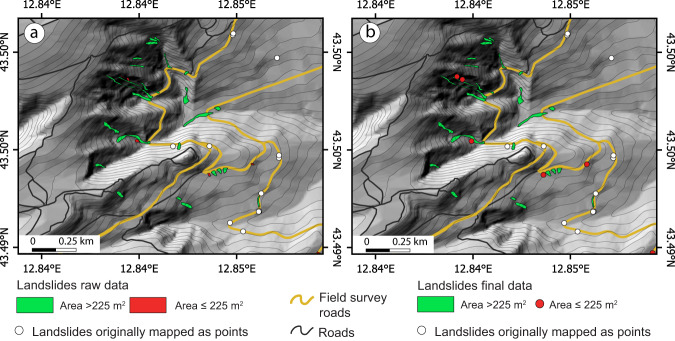
Excerpts of the E-LIM at the publication scale (1: 15 000). (**a**) Original inventory where landslides are mapped as polygons if estimated larger than a few tens of square metres (raw data). Green polygons are landslides larger than 225 m^2^, red polygons are landslides smaller than 225 m^2^. (**b**) Inventory after post-processing. Green polygons are landslides larger than 225 m^2^, red points are landslides formerly represented as polygons but below the size threshold of 225 m^2^ (red polygons in (**a**). In both panels: white points are landslides originally mapped as points, black lines are roads, yellow lines are roads used during the field survey. Base map: TINItaly DEM^40^ and derived contours at 10 m equidistance. Roads from OpenStreetMap^35^.

To take into account mapping errors in the final map, we estimated that a consulting scale of 1:15 000 would be consistently supported throughout the map. Such a decision has a direct impact on the minimum size of the landslide represented as a polygon. In the final inventory, all the landslides larger than 225 m^2^, i.e. a square of 15 m per side (1 mm per side at the scale of the map) were represented as polygons, whereas the ones below this threshold were represented as point features. However, since in the field it is impossible to estimate landslide size with such a degree of accuracy, during the reconnaissance field survey, landslides estimated larger than a few tens of square metres (i.e. far below the threshold of 225 m^2^) were mapped as polygons to prevent loss of information in the final inventory (E-LIM raw data in Fig. 2). Later, in the office, landslides that were originally mapped as polygons but showing an area smaller than the 225 m^2^ were transformed into points (i.e. the centroid of the polygon), preserving the area value in the attribute table. On the contrary, landslides that were originally represented as points have no area value (Fig. 3).

After completion of reconnaissance field survey activities, the 8 E-LIMs for the 8 sectors were checked in the office by the expert geomorphologists by verifying the location, classification, and delineation of the landslide borders using the field pictures (Fig. 2). For each landslide, where available, a maximum of two pictures were selected and reported in the attribute table. Later, the checked E-LIMs were merged in a single database (Fig. 2) and a topological check was carried out using ArcGIS^37^ tools to avoid overlapping polygons. Eventually, technical validation (Fig. 2) was carried out by all the 5 geomorphologists who led the field activities. Results are presented in the Technical Validation section.

## Data Records

All collected data were stored in a repository and are publicly available for download at 10.6084/m9.figshare.21981842.v1^34^. In the archive, a total of 5 shapefiles and 942 field pictures are stored (Table 2).

**Table 2:** Description of the files contained in the public repository.

File/folder name	Format	Description
AOI.shp	vector	Polygon of the AOI.
ELIM_polygon.shp	vector	Polygon map of landslides larger than 225 m^2^
ELIM_point.shp	vector	Point map of landslides originally mapped as points, and landslide smaller than 225 m^2^
ELIM_polygon_raw_data.shp	vector	Map of landslides originally mapped as polygons (raw data)
ELIM_point_raw_data.shp	vector	Map of landslides originally mapped as points (raw data)
Selected_pictures	folder	Folder containing all geotagged field pictures selected and listed in the attribute table of the E-LIM.

Table 3 reports the structure of the attribute table associated with both point and polygon layers (second and third rows of Table 2). Table 4 describes the possible values of the field “Cod_Type” of layers listed at the second and third rows of Table 2.

**Table 3:** Table structure of point and polygon layers.

Field name	Type	Description
ID	Integer	Unique numeric identifier
Date	Date	Date of the field observation
Zone	String	Name of the sector
Movement	String	Type of movement
Material	String	Type of involved material
Cod_Type	String	Code associated to the landslide classification (movement and material)
Description	String	Extended description of landslide classification
Pic_1	String	File name of first selected field picture
Pic_2	String	File name of second selected field picture
Shape_length	Double	Perimeter length in metres
Shape_area	Double	Area in square metres

**Table 4:** Dictionary of the field “Code_type” of both point and polygon layers.

Code_Type	Description
es	Earth slide
esf	Earth slide flow
ef	Earth flow
ds	Debris slide
dsf	Debris slide flow
df	Debris flow
rs	Rock slide
rf	Rock fall
wes	Widespread earth slide
wdf	Widespread debris flow
wds	Widespread debris slide

The inventory covers an area of 550 km^2^ and includes 1,687 landslides, corresponding to an average density of about 3.1 landslides per square kilometre. Landslide size (Landslide Area, A_L_ in m^2^) is in the range ~1 m^2^ < A_L_ < 5.7 × 10^4^ m^2^. Overall, landslides cover an area of 1.1 km^2^ (Table 5), which represents 0.2% of the AOI.

**Table 5:** Count of features (ply = polygons; pnt = points) in the final inventory based on landslide movement and material.

	Fall		Flow		Slide-flow		Slide		Total	
	*#Ply*	*#Pnt*	*#Ply*	*#Pnt*	*#Ply*	*#Pnt*	*#Ply*	*#Pnt*	*#Ply*	*#Pnt*
**Rock**	4	14	—	—	—	—	3	3	7	17
**Debris**	—	—	241	63	109	23	122	110	472	196
**Earth**	—	—	30	35	60	28	162	680	252	743
**Total**	4	14	271	98	169	51	287	793	731	956

**Fig. 4:**
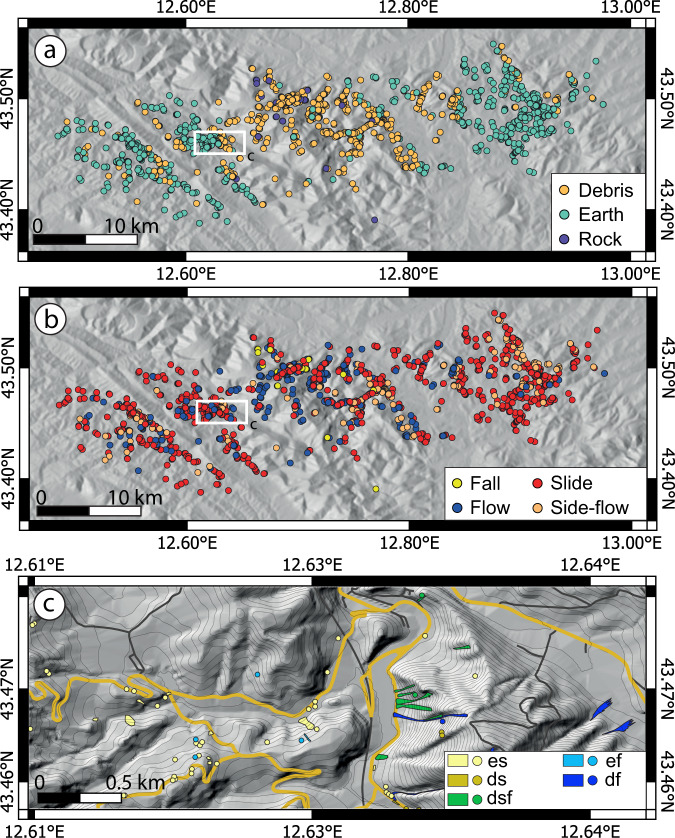
Location of landslides classified based on material involved (**a**) and type of movement (**b**). Panel (**c**) shows an excerpt of the final E-LIM at the publication scale (1:15 000). Base map: TINItaly DEM^40^ and derived contours at 10 m equidistance. Roads from OpenStreetMap^35^, colour-coded as in Fig. 3.

In the raw inventory (Fig. 2), a total of 1243 landslides were mapped as polygons directly in the field, whereas the remaining 444 landslides were mapped as points (i.e. without information on landslide area). After the post-processing activities, 512 polygons were transformed into points because their area was below the 225 m^2^ size threshold. Therefore, in the final inventory, 731 landslides are represented as polygons and 956 as points, ~54% of which retained the original area value (Table 5). Table 6 summarises the number (N_L_) and area (A_L_) of landslides according to the material involved and the type of movement. Figure 4 shows the spatial distribution of landslides according to material (Fig. 4a) and type of movement (Fig. 4b). Figure 4c shows a detail of the final E-LIM.

**Table 6:** Number and total area of landslide types represented in the event landslide inventory map.

	Fall		Flow		Slide-flow		Slide		Total	
	*N*_*L*_	*A*_*L*_ (*m*^2^)	*N*_*L*_	*A*_*L*_ (*m*^2^)	*N*_*L*_	*A*_*L*_ (*m*^2^)	*N*_*L*_	*A*_*L*_ (*m*^2^)	*N*_*L*_	*A*_*L*_ (*m*^2^)
**Rock**	18	5.1 × 10^3^	—	—	—	—	6	6.9 × 10^3^	24	1.2 × 10^4^
**Debris**	—	—	304	6.0 × 10^5^	132	1.9 × 10^5^	232	1.1 × 10^5^	668	9.0 × 10^5^
**Earth**	—	—	65	2.2 × 10^4^	88	4.9 × 10^4^	842	1.4 × 10^5^	995	2.1 × 10^5^
**Total**	18	5.1 × 10^3^	369	6.2 × 10^5^	220	2.4 × 10^5^	1080	2.5 × 10^5^	1687	1.1 × 10^6^

N_L_: number of landslides; A_L_: landslide area in square metres.

**Fig. 5:**
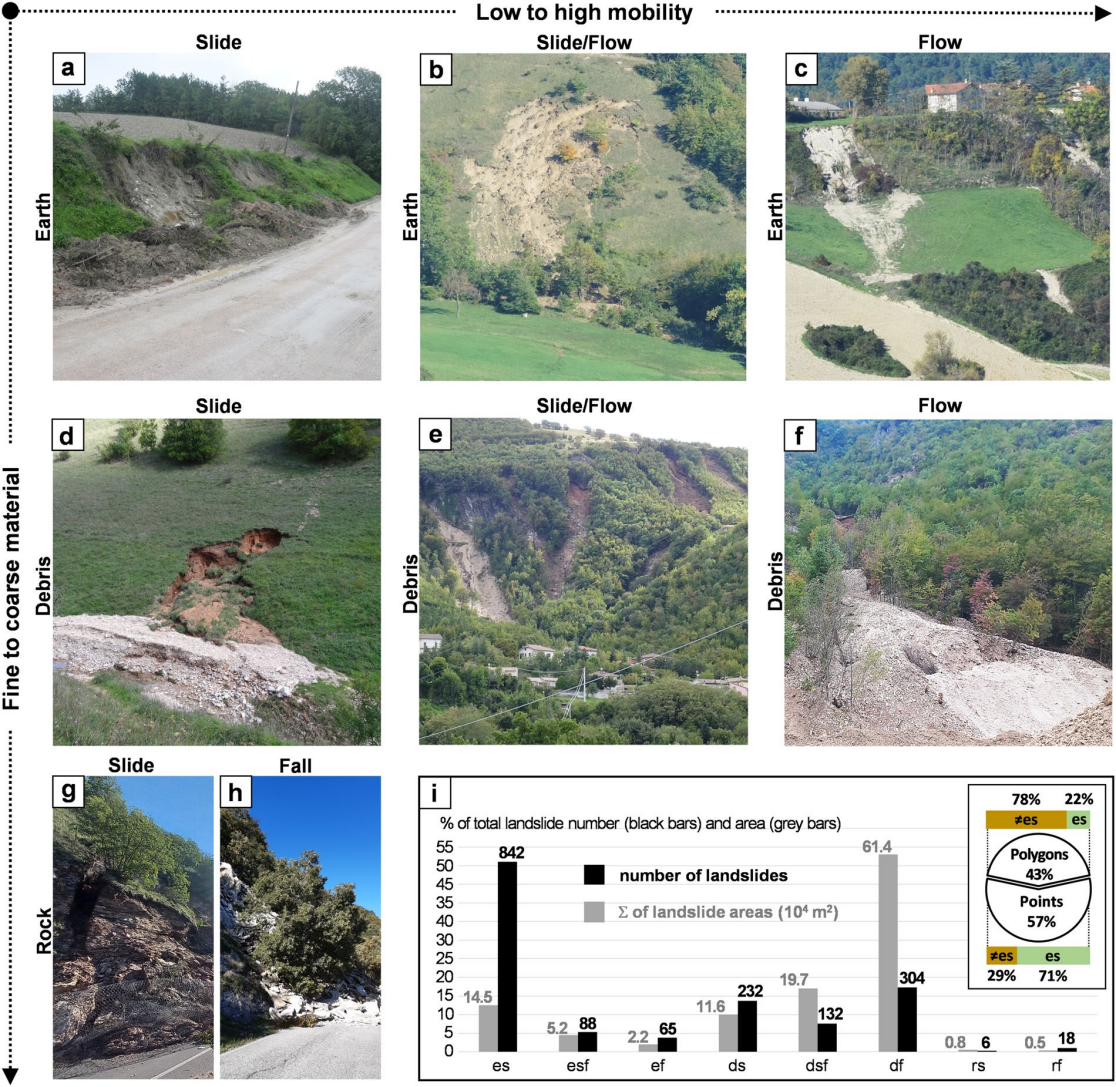
Examples of the triggered landslides by material (columns) and type of movement (rows). (i) Barplot reporting the number and total area in 10^4^ m^2^ of each landslide type and pie chart showing the relative abundance of points and polygons in the final E-LIM also based on their classification. Labels in the x-axis of the bar plot and the pie chart are the same as in Table 4.

In Fig. 5, pictures taken in the field provide examples of the different landslide types that occurred in the event. Inspection of the figure reveals that both channelled and un-channelled movements occurred, showing different degrees of mobility, and that type of material included rock, earth, and debris. Figure 5i shows the relative abundance in terms of number and total area for each landslide type, according to Table 5.

## Technical Validation

After the preparation of the final version of the E-LIM, a technical validation process was carried out by the 5 expert geomorphologists, to check: (i) the database compilation completeness and integrity; (ii) the correspondence between the photos taken in the field and the observed landslides; (iii) the geographic accuracy (*sensu* Guzzetti *et al*., 2012), i.e., size, location and shape of the individual landslides; (iv) the thematic accuracy (*sensu* Guzzetti *et al*., 2012), i.e., landslide classification; (v) the actual amount of territory observed from the roads used during the reconnaissance field survey.

Activities to validate the database completeness and correctness as well as the estimation of the amount of territory actually observed were carried out over the entire dataset/territory. All the other activities were carried out on a random sample of ~10% of the features originally mapped as polygons.

To check the completeness of the database compilation, a systematic check was carried out over the entire database tables to detect empty records (i.e. completeness) or typing errors (i.e. referential integrity) compared to dictionaries (e.g. Table 4).

**Fig. 6:**
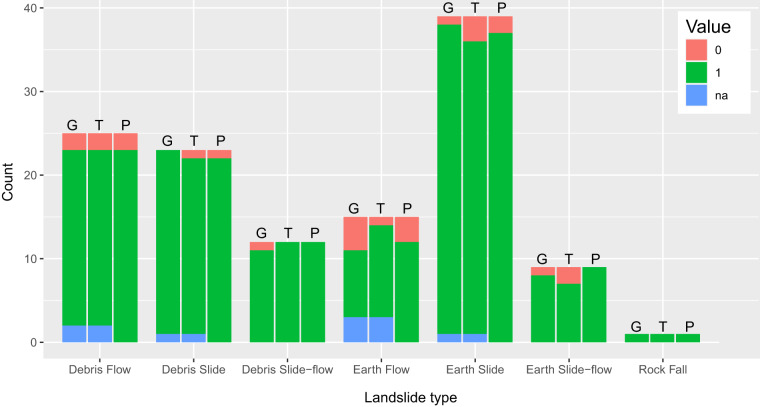
Results of the technical validation carried out on 10% of landslides originally mapped as polygons. In the figure: G, geographic accuracy; T, thematic accuracy; P, field picture check. Results are disaggregated based on landslide type. In the legend: na, not applicable, 0, unacceptable, 1, acceptable.

The correspondence between the photos taken in the field and the landslides observed was assigned a binary score (0,1): 0 in case of pictures not referring to the mapped landslide indicated in the database record; 1 in case of pictures corresponding to the mapped landslide indicated in the database record (Fig. 6).

For all the landslides in which records have at least one picture assigned, it was possible to evaluate the geographic and thematic accuracy. The geographic accuracy refers to how the mapping matches the ground truth (i.e field pictures) in terms of location, size and shape. Therefore, it was possible to evaluate the geographic accuracy only for landslides originally mapped as polygons (i.e. “raw data” in Fig. 2). Firstly, the polygons of the selected landslides were buffered with a distance of 7.5 m per side to take into account a total 15 m error compatible with the scale of the final map. Then, a binary score was assigned to each record by comparing the mapped polygon and the field picture(s). A value of 0 was assigned if the landslide was not completely contained in the buffer (i.e., the mapping was considered inaccurate). Otherwise the mapping was considered geographically accurate (value 1 was assigned).

Similarly to the geographic accuracy, each landslide polygon with at least one selected picture was assigned a binary score based on the correspondence between the assigned classification in the database record and the geomorphological evidences portrayed in the selected picture(s). Mapping was considered thematically inaccurate (value = 0) if the classification was considered not correct. On the contrary it was assigned a value of 1 (i.e. mapping was considered thematically accurate).

Finally, all landslides without an associated picture were assigned the value “na” for both the geographic and thematic accuracy evaluation, as it was impossible to compare the mapping to a field picture (Fig. 6).

In total, we randomly selected 150 polygons, 26 of which had no associated picture. For the remaining 124 features (10% of landslides originally mapped as polygons) shown in Fig. 6, 9 exhibited an unacceptable geographic accuracy compared to the scale of the final map (7.2%), 9 showed an unacceptable thematic accuracy and 8 were associated with an incorrect field picture (6.4%). Figure 6 resumes the outcomes of the technical validation activities carried out for the geographic accuracy, thematic accuracy, and field pictures (G, T, and P in Fig. 6 respectively) according to landslide types.

Checks carried out on the entire database showed both no empty records or referential integrity issues.

Finally, we performed a visibility analysis exploiting the r.survey tool published by Bornaetxea *et al*.^39^ to estimate the amount of territory that has been observed from the roads that were used during the field survey (e.g. Figs. 3, 4c). The analysis requires as input an average height of the observer(s) which was set to 1.7 m, a DEM (we used the TINItaly DEM at 10 m GSD published by Tarquini *et al*.^40^), and an equidistant step along the road network, which was set to 50 metres.

**Fig. 7:**
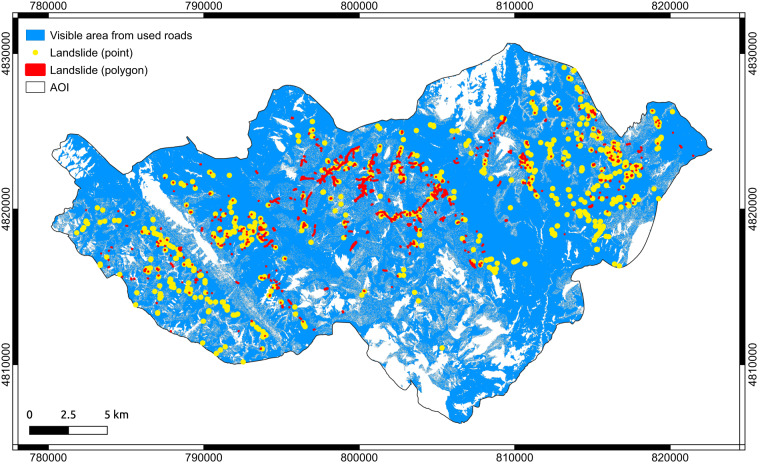
Results of the visibility analysis. In blue the territory visible from the used roads. Red polygons and yellow dots: landslides represented as polygons and points respectively. Coordinates EPSG: 32632.

The visibility analysis revealed that a total 112 km^2^ of the AOI (i.e. 20.2%) could not be inspected due to accessibility/visibility constraints along the road network (Fig. 7). Among the different outputs of the tool, we decided to use a synthetic map that represents the overall territory visible from the used roads. Inspection of the figure reveals that the territory can be classified in areas that are not visible (continuous white areas), areas poorly visible (salt and pepper texture), areas visible (continuous blue). It is worth noting that no landslides were mapped in the continuous white areas, which reinforces the results of the good geographic accuracy that results from the technical validation. In such areas our inventory should be considered as “no data” rather than landslide-free area. In addition, it must be noted that since the inventory has been carried out for a portion of the overall landslide event (i.e., the AOI), the landslide inventory map presented in this paper is not a landslide inventory map of the entire event. On the other hand, we maintain it is a representative and as complete as possible snapshot of the landslide distribution and types triggered on both natural and artificial slopes by the extreme rainfall event that hit Marche-Umbria regions on 15^th^ September 2022.

## Data Availability

The Authors declare that no custom code was used.
